# Phenotypic Variation Analysis and Excellent Clone Selection of *Alnus cremastogyne* from Different Provenances

**DOI:** 10.3390/plants12183259

**Published:** 2023-09-13

**Authors:** Yue Zheng, Maosong Feng, Xue Li, Xingyan Huang, Gang Chen, Wenyu Bai, Xueju Xu, Jiayi Li, Xiaohong Li, Bin Leng, Hao Sun, Chunyan He, Yunjie Chen

**Affiliations:** 1 College of Forest, Sichuan Agricultural University, Chengdu 611130, China; 2021304055@stu.sicau.edu.cn (Y.Z.); lx1284581938@163.com (X.L.); hxy@sicau.edu.cn (X.H.); g.chen@sicau.edu.cn (G.C.); 71387@sicau.edu.cn (W.B.); 17623666514@163.com (X.X.); l1113145275@163.com (J.L.); a15680753093@outlook.com (X.L.); lingbin410@gmail.com (B.L.); 15680832195@163.com (H.S.); hcy01160407@163.com (C.H.); cyj199806@163.com (Y.C.); 2 National Forestry and Grassland Administration Key Laboratory of Forest Resources Conservation and Ecological Safety on the Upper Reaches of the Yangtze River and Forestry Ecological Engineering in the Upper Reaches of the Yangtze River Key Laboratory of Sichuan Province, Chengdu 611130, China

**Keywords:** *Alnus cremastogyne*, phenotypic traits, phenotypic variation, excellent clone

## Abstract

*Alnus cremastogyne* is a rapidly growing broad-leaved tree species that is widely distributed in southwest China. It has a significant economic and ecological value. However, with the expansion of the planting area, the influence of phenotypic variation and differentiation on *Alnus cremastogyne* has increased, resulting in a continuous decline in its genetic quality. Therefore, it is crucial to investigate the phenotypic variation of *Alnus cremastogyne* and select excellent breeding materials for genetic improvement. Herein, four growth-related phenotypic traits (diameter at breast height, the height of trees, volume, height under the branches) and twelve reproductive-related phenotypic traits (fresh weight of single cone, dry weight of single cone, seed weight per plant, thousand kernel weight, cone length, cone width, cone length × cone width, fruit shape index, seed rate, germination rate, germination potential, germination index) of 40 clones from four provenances were measured and analyzed. The phenotypic variation was comprehensively evaluated by correlation analysis, principal component analysis and cluster analysis, and excellent clones were selected as breeding materials. The results revealed that there were abundant phenotypic traits variations among and within provenances. Most of the phenotypic traits were highly significant differences (*p* < 0.01) among provenances. The phenotypic variation among provenances (26.36%) was greater than that of within provenances clones (24.80%). The average phenotypic differentiation coefficient was accounted for 52.61% among provenances, indicating that the phenotypic variation mainly came from among provenances. The coefficient of variation ranged from 9.41% (fruit shape index) to 97.19% (seed weight per plant), and the repeatability ranged from 0.36 (volume) to 0.77 (cone width). Correlation analysis revealed a significantly positive correlation among most phenotypic traits. In principal component analysis, the cumulative contribution rate of the first three principal components was 79.18%, representing the main information on the measured phenotypic traits. The cluster analysis revealed four groups for the 40 clones. Group I and group II exhibited better performance phenotypic traits as compared with group III and group IV. In addition, the four groups are not clearly clustered following the distance from the provenance. Employing the multi-trait comprehensive evaluation method, 12 excellent clones were selected, and the average genetic gain for each phenotypic trait ranged from 4.78% (diameter at breast height) to 32.05% (dry weight of single cone). These selected excellent clones can serve as candidate materials for the improvement and transformation of *Alnus cremastogyne* seed orchards. In addition, this study can also provide a theoretical foundation for the genetic improvement, breeding, and clone selection of *Alnus cremastogyne.*

## 1. Introduction

*Alnus cremastogyne*, a deciduous tree, belonging to the genus *Alnus* in the family Betulaceae, is an endemic species among the 11 *Alnus* species found in China [1]. *A. cremastogyne* is native to the Sichuan Basin and its surrounding areas. It has been widely introduced and cultivated in the middle and lower reaches of the Yangtze River Basin since the 1960s [2]. Due to its rapid growth, it plays a crucial role in the establishment of short-cycle industrial raw material forests. It is rich in cellulose, making it an excellent raw material for producing high-quality paper [3]. As a non-leguminous nitrogen-fixing tree, *A. cremastogyne* has a well-developed root system with nodules that efficiently fix free nitrogen from the atmosphere, thereby enhancing soil fertility [4]. In addition, it has been extensively employed as a raw material in the manufacturing of plywood, musical instruments, furniture, and other related products for its excellent wood properties [5].

Phenotypic variation in forest trees is a crucial manifestation of genetic diversity, adaptability, and evolution [6]. Phenotypic variation is a significant outcome arising from the combined influence of genetic and environmental factors. The tree growth reflects the adaptation of genotypes to environmental changes, irreversible changes in long-term stress selection, and emergence of new phenotypes following stable inheritance [7]. Therefore, the investigation of phenotypic variations is beneficial to unraveling the intricate mechanisms of gene–environment interactions. It also can provide crucial insights for optimizing and enhancing the germplasm resources of forest tree species [8]. In addition, the phenotypic variation of forest trees not only reflects the dispersion pattern of different populations and species, but can also provide a more comprehensive understanding of the germplasm diversity of breeding materials by analyzing the phenotypic variation of forest trees. Breeders conduct targeted breeding work based on the characteristics of phenotypic variations in various germplasm resources, for identifying high-quality genetic trait breeding materials and genetic improvement [9]. Various research methods have been employed to study the genetic variation of trees, including phenotypic trait measurement, cytological markers, biochemical markers, and molecular markers [10,11,12]. Among these methods, the study of phenotypic trait measurement has the advantages of quickness and simplicity. At present, phenotypic variation is widely studied in various plant species to investigate genetic diversity [13,14,15]. Thus, studying phenotypic variation provides a more comprehensive understanding of the genetic variation in breeding materials, and can also facilitate genetic improvement of trees based on the characteristics of genetic variation.

Excellent clone selection is an important basis for forest tree genetics and breeding [16]. Due to the long genetic breeding cycle of forest trees, the selection of excellent clones is a basic approach utilized in genetic improvement. Selecting excellent clones with desirable traits in the early stages of tree growth can expedite subsequent breeding processes. It also effectively reduces the generation interval in reproduction, thereby shortening the breeding cycle in tree breeding programs. Additionally, these selected excellent clones, through asexual reproduction, stabilize the genetic advantages of these individuals and pass them on to the offspring. This approach can result in higher genetic gains. Due to the combination of individual genetic factors and external environmental influences, trees possess abundant genetic variation. Analyzing this genetic variation can facilitate the identification and selection of excellent clones, which is highly valuable for genetic improvement of trees [17,18,19]. Therefore, revealing the variation patterns of phenotypic traits is the basis of scientific breeding strategy. Moreover, provenances tests are also considered as one of the important means to select excellent breeding materials [20]. Provenance tests also can reveal the environmental and genetic mechanism of variation, so that clones can be targeted and selected as breeding materials to improve the genetic gain of offspring [21]. Currently, researchers have conducted provenance tests to analyze phenotypic variations and to select excellent clones of tree species such as *Juglans regia* [22], *Eucalyptus urophylla* [23], and *Larix kaempferi* [24], etc.

As an important economic tree species, extensive research has been conducted on the *Alnus* species. These studies have mainly focused on their gene sequences [25,26,27], the antioxidant and pharmacological properties of extracts [28,29,30], nitrogen fixation of rhizobia [31,32], photosynthetic physiology [33,34], etc. In recent years, extensive efforts have been made across different regions of China to promote the establishment of fast-growing and high-yielding forests, which requires a substantial supply of high-quality *A. cremastogyne* seeds for afforestation. However, most of the used *A. cremastogyne* seeds are collected in the wild, lacking a proper selection. Hence, there is an urgent need to improve breeding materials’ quality through genetic improvement. Unfortunately, the research on the genetic improvement of *A. cremastogyne* is mainly focused on conventional breeding, and the study of phenotypic variation remains somewhat inadequate. In previous studies, Chen et al. [35] studied the phenotypic characteristics of *A. cremastogyne* from different provenances and found that there were extremely rich phenotypic variations among and within provenances. Moreover, there were highly significant differences among different provenances for the tree height and volume of *A. cremastogyne*; in addition, the interaction between provenance × location was significant [36]. These findings indicated that there was an interaction effect between provenance and environment, which provided a reliable genetic background for provenance selection of *A. cremastogyne*. Subsequently, Chen et al. [37] found that there were extensive variations in fruiting and seed traits among different clones in the study of *A. cremastogyne*. The above mentioned studies on the genetic variation of *A. cremastogyne* were based on the measurement and analysis of phenotypic traits in growth, cone, seed, germination, and other traits, while rarely considering the association of several phenotypic traits. It is difficult to clarify this genetic variation in detail and accurately, which hinders the further exploration and utilization of excellent germplasm resources for *A. cremastogyne*.

In this study, 16 phenotypic traits (growth-related phenotypic traits and reproductive -related phenotypic traits) of 40 *A. cremastogyne* clones from four provenances were measured and analyzed. The main purposes of this study were: (a) to analyze the variation characteristics of phenotypic traits and determine the differentiation of phenotypic traits; (b) to select excellent clones as breeding materials for genetic improvement in *A. cremastogyne* seed orchard based on phenotypic variation characteristics. This study will provide a theoretical basis for genetic improvement and breeding of *A. cremastogyne*.

## 2. Results

### 2.1. Variation Analysis of Phenotypic Traits among and within Provenances of 40 A. cremastogyne Clones from 4 Provenances

**Figure 1:**
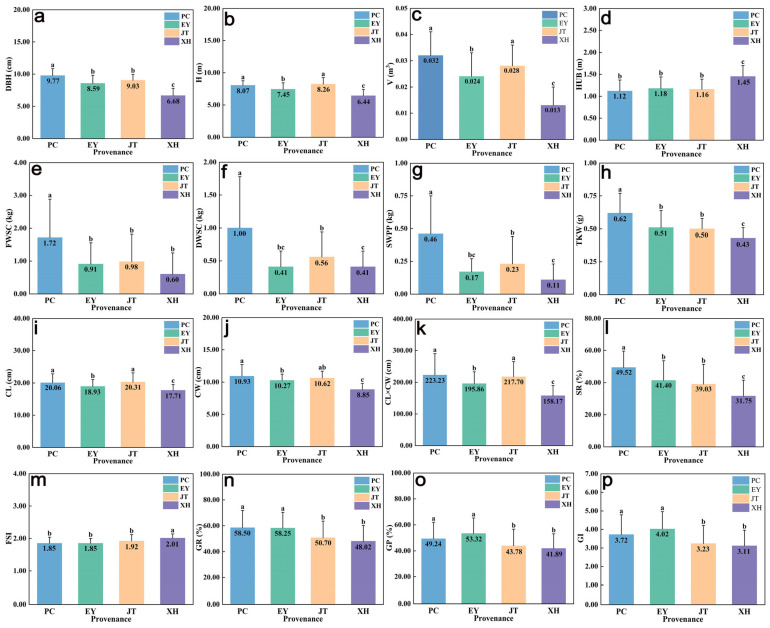
Multiple comparison of the average values of phenotypic traits of 4 provenances of *A. cremastogyne* (**a**–**p**). In the bar chart. The blue, green, yellow and purple rectangular bars are respectively represented as PC: Pingchang County; EY: Enyang District; JT: Jintang County; XH: Xuanhan County; the same letter on the error bars indicates no significant difference, Numbers in bars represent average values. The abbreviation of phenotypic traits is shown in Table 1.

The variance analysis considered 16 phenotypic traits of 40 *A. cremastogyne* clones from 4 provenances of Pingchang County (PC), Enyang District (EY), Jintang County (JT), and Xuanhan County (XH) in Sichuan Province in the seed orchard. The results of variance analysis showed that highly significant differences (*p* < 0.01) were observed in all phenotypic traits among provenances. Except for the volume, other phenotypic traits were highly significant differences (*p* < 0.01) or significant differences (*p* < 0.05) within provenances (Table 1). The phenotypic traits of different provenances were tested by Duncan’s multiple comparison test (Figure 1). Significant differences (*p* < 0.05) were observed in the phenotypic traits across the 4 provenances, indicating extensive variation. As for growth traits, the PC provenance had the largest DBH (9.77 cm) and V (0.032 m³), while the JT provenance had the largest H (8.07 m). Conversely, the XH provenance had the smallest DBH (6.68 cm), H (6.44 m), and V (0.013 m³). Concerning cone and seed traits, the PC provenance had the largest FWSC (1.72 kg), DWSC (1.00 kg), SWPP (0.46 kg), TKW (0.62 g), and SR (49.52%), whereas the XH provenance had the smallest FWSC (0.60 kg), DWSC (0.41 kg), SWPP (0.11 kg), TKW (0.43 g), and SR (31.75%). They were 2.9 times, 2.4 times, 4.1 times, 1.4 times, and 1.6 times, respectively, greater than those of the XH provenance. No significant differences on GR, GP, and GI between the PC and EY provenance were observed regarding germination traits. However, significant differences (*p* < 0.05) were observed between the PC, JT, and XH provenance. Additionally, there were no significant differences in GR, GP, and GI between JT and XH provenance.

### 2.2. Phenotypic Differentiation among Provenances

**Figure 2:**
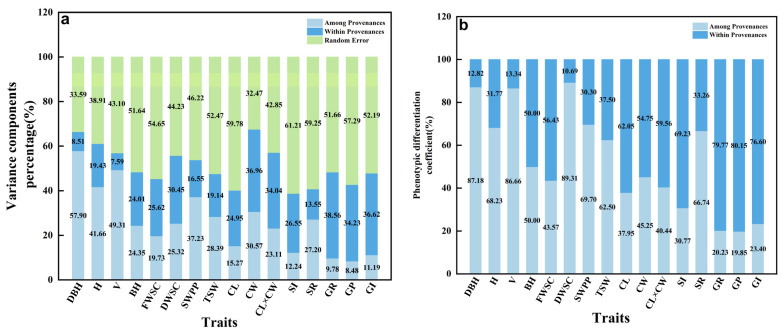
Variance components percentage of 16 phenotypic traits (**a**); phenotypic differentiation coefficient among and within provenances (**b**). The abbreviation of phenotypic traits is shown in Table 1.

**Figure 3:**
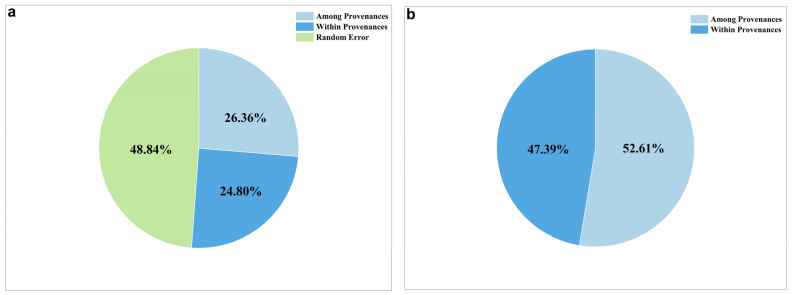
The average variance component proportion of 16 phenotypic traits (**a**); proportion of average phenotypic differentiation coefficient among and within provenances (**b**).

The variance components and phenotypic differentiation coefficients of 16 phenotypic traits were calculated according to the results of nested variance analysis. The variance component of 16 phenotypic traits are shown in Figure 2a, which ranged from 8.48% (GP) to 57.90% (DBH) among provenances, and ranged from 7.59% (V) to 38.56% (GR) within provenances. The average variance component among provenances was 26.36% of the total variation, and that within provenances accounted for 24.80% of the total variation (Figure 3a). The average variance components among and within provenances accounted for 26.36% and 24.80% of the total variation. The phenotypic differentiation coefficient ranged from 19.85% to 89.31% (Figure 2b). Among them, the largest phenotypic differentiation coefficient was observed for DWSC (89.31%), demonstrating that it had the highest degree of differentiation among all traits. Additionally, the phenotypic differentiation coefficients of DBH, H, V, DWSC, SWPP, TSW, and SR exceeded 50%, indicating significant differentiation among provenances of these phenotypic traits. In contrast, the smallest phenotypic differentiation coefficient was observed for GP (19.85%), and thereby it was the most stable among all traits. At the same time, compared with the phenotypic differentiation coefficients of other traits, the phenotypic differentiation coefficients of SI (30.77%) and GR (20.23%) were also low, suggesting that they were less differentiated and stable among provenances. The average phenotypic differentiation coefficient accounted for 52.61% among provenances, indicating that the phenotypic variation of *A. cremastogyne* was mainly seen among provenances (Figure 3b).

### 2.3. Variation Degree and Genetic Parameters of Phenotypic Traits

The average values, minimum values (min), maximum values (max), range, and standard deviation (SD) of 16 phenotypic traits were summarized in Table 2. The max values recorded for FWSC, DWSC, and SWPP were 3.86, 2.84, and 0.96, respectively, and the min ones were 0.19, 0.11, and 0.06, respectively. The max values were 20.32, 25.82, and 16.00 times greater than the corresponding min values, demonstrating substantial variation in these traits as compared to others.

**Figure 4:**
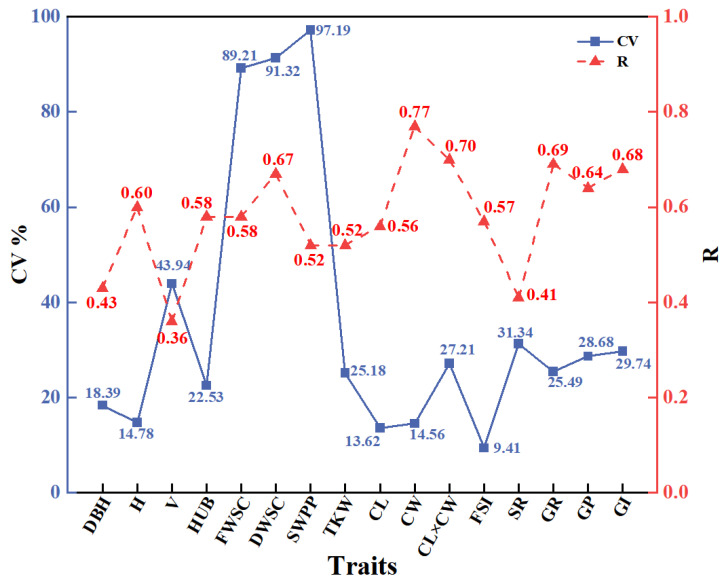
Coefficient of variation (CV) and repeatability(R) of phenotypic traits. The abbreviation of phenotypic traits is shown in Table 1.

Coefficient of variation (CV) and repeatability are commonly used as two important genetic parameters, representing the dispersion and genetic stability of phenotypic traits. Figure 4 illustrating the CV and repeatability of each phenotypic trait, revealing a high overall level of variability in the phenotypic traits of *A. cremastogyne*, with abundant variation among the different phenotypic traits. The coefficients of variation for each phenotypic trait ranged from 9.41% to 97.19%. Among them, the highest coefficient of variation was 97.19% for SWPP, and the lowest was 9.41% for FSI. Additionally, the SWPP, DWSC, and FWSC exhibited relatively large CV values of 97.19%, 91.32%, and 89.21%, respectively. On the other hand, the DBH, H, CL, CW, and FSI had relatively smaller CV values of 18.39%, 14.78%, 13.62%, 14.56%, and 9.41%, respectively. The repeatability of the 16 phenotypic traits in *A. cremastogyne* varied from 0.36 to 0.77. The highest repeatability value was 0.77 for CW, while the lowest was 0.36 for V. Among the tested phenotypic traits, the repeatability values for DBH (0.43), V (0.36), and SR (0.41) were less than 0.5. However, the repeatability values for the remaining phenotypic traits were all greater than 0.5. The high coefficients of variation and repeatability reflected the immense potential for genetic improvement.

### 2.4. Correlation Analysis of Phenotypic Traits

**Figure 5:**
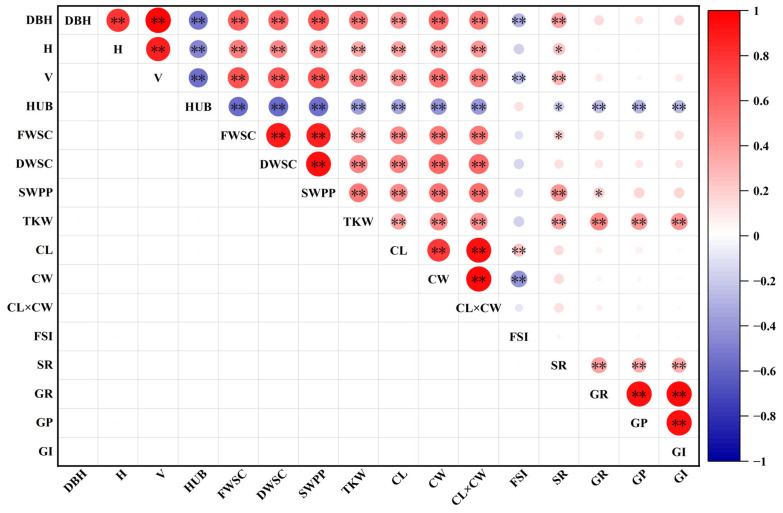
Correlation analysis of phenotypic traits. In the figure, the larger the circle, the deeper the color represents the stronger the correlation, *: *p* < 0.05, **: *p* < 0.01. The abbreviation of phenotypic traits is shown in Table 1.

The correlation analysis results of the phenotypic traits are depicted in Figure 5. There were significantly positive (*p* < 0.05) correlation among most phenotypic traits. The growth-related phenotypic traits, DBH, H, and V demonstrated highly significant (*p* < 0.01) positive correlations, indicating a strong association among these three growth traits. Remarkably, the correlation coefficient between DBH and V stood out as the highest among all phenotypic traits, further highlighting the strong relationship between DBH and V. In contrast, HUB exhibited no significant correlation with FSI, and was negatively correlated with various phenotypic traits. In addition, DBH, H, and V were significantly and positively correlated with FWSC, DWSC, SWPP, TKW, CL, CW, CL × CW, and SR, respectively. Regarding reproductive-related phenotypic traits, FWSC, DWSC, SWPP, CL, CW, and CL × CW were also significantly and positively correlated. Additionally, GR, GP, and GI showed significant positive correlations, as well as with TKW. These correlations among traits highlight the potential for evaluating and selecting exceptional genotypes of *A. cremastogyne* based on their growth characteristics, fruit traits, seed traits, and germination performance.

### 2.5. Principal Component Analysis (PCA) of Phenotypic Traits

**Figure 6:**
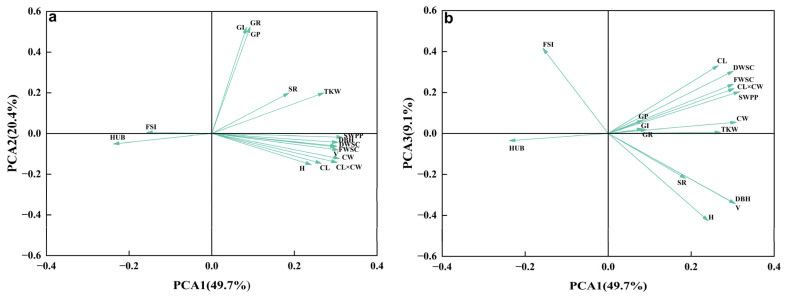
PCA of phenotypic traits of *A. cremastogyne*. The projection of the load of 16 phenotypic traits of *A. cremastogyne* on PCA1 and PCA2 (**a**); the projection of the load of 16 phenotypic traits of *A. cremastogyne* on PCA1 and PCA3 (**b**). The abbreviation of phenotypic traits is shown in Table 1.

The PCA was conducted on 16 phenotypic traits of *A. cremastogyne* (Table 3). Three principal components were extracted with eigenvalues greater than 1, capturing a cumulative contribution rate of 79.18% of the variance. The first principal component (Y_1_), with an eigenvalue of 7.95, was 49.69% of the variance. DBH (0.86), H (0.68), V (0.86), FWSC (0.85), DWSC (0.85), SWPP (0.89), TSW (0.77), CL (0.75), and CW (0.87) exhibited the highest eigenvalues. Furthermore, this had the most significant impact on the first principal component. This indicated that the first principal component primarily represented the information related to growth, cones, and seeds. The second principal component (Y_2_) had an eigenvalue of 3.26, with 20.36% of the variance. Among its contributing traits, GR (0.95), GP (0.94), and GI (0.94) had the highest eigenvalues, suggesting that the second principal component mainly represented information related to germination characteristics. The third principal component (Y_3_) had an eigenvalue of 1.46, was 9.13% of the variance. HUB (−0.04) and FSI (0.50) had the highest eigenvalues, suggesting that the third principal component primarily represented information regarding the seed yield in cones. Importantly, the eigenvalue of HUB was negative, indicating an inverse relationship between the HUB value and the score. In the principal component analysis loadings plot (Figure 6a,b), the variables of DBH, H, V, FWSC, DWSC, SWPP, TSW, CL, and CW for the *A. cremastogyne* were consistent with PCA1 principal component values. The variables GR, GP, and GI were consistent with PCA2 principal component values. HUB and FSI were relatively close to PCA3 principal component values.

**Figure 7:**
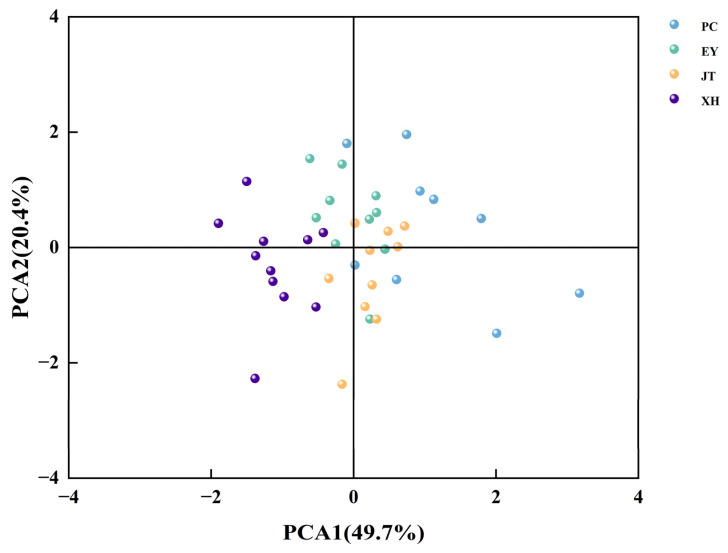
The scatter plot of phenotypic traits of 40 *A. cremastogyne* clones from 4 provenances based on the PCA1 and PCA2. The abbreviation of phenotypic traits is shown in Table 1.

The PCA scatter plot was constructed with PCA1 on the X-axis and PCA2 on the Y-axis (Figure 7). The geographic distribution of different provenances of *A. cremastogyne* significantly influenced the phenotypic traits of the species. Its impact on growth, cone, and seed traits (PCA1) were greater than that on germination traits (PCA2). PC and XH provenances of *A. cremastogyne* were highly influenced by the geographic factors in terms of their phenotypic traits. However, the phenotypic traits of EY and JT provenances were less affected by geographic factors. EY and JT provenances had higher degrees of consistency in their phenotypic traits, while PC and XH provenances showed a larger variation in phenotypic traits.

### 2.6. Cluster Analysis of Clone

**Figure 8:**
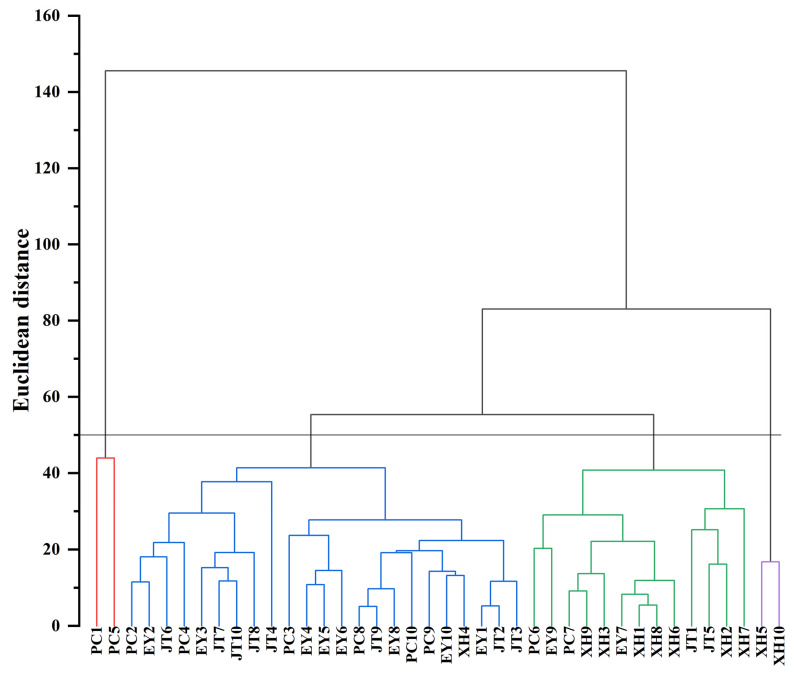
Cluster analysis figure of 16 phenotypic traits of 40 *A. cremastogyne* clones based on Euclidean distance from 4 provenances. The clone number abbreviation is shown in Figure 1.

The 40 clones from 4 provenances were clustered using the inter-group connection method based on Euclidean distance. The results are displayed in Figure 8. With a Euclidean distance threshold of 50, the 40 clones were classified into four groups. The average value of each phenotypic trait was calculated for the four groups (Table 4). The group I, with two clones (PC1 and PC5) from the PC provenance, represented 5.00% of the total clones. This group had the largest average value of DBH (10.48), H (8.17), V (0.04), FWSC (3.31), DWSC (2.09), SWPP (0.84), TSW (0.69), CL (24.03), CW (13.91), and CL × CW (335.28). Group II comprised 23 clones from the PC, EY, and JT provenance, accounting for 57.50%, which had the highest average value of FSI (1.91) and GP (50.63). Group III comprised 13 clones primarily sourced from the XH provenance, accounting for 32.50% of the total. The main characteristics of this group, and in this group only, was the largest average value of FSI (2.10). Group IV included two XH5 and XH10 clones originating from the XH provenance. These clones accounted for 5.00% of the total. In this group, the average value of HUB (1.58) and GR (58.85) were the largest, and the average value of FSI (2.10), GP (50.17) and GI (3.62) were relatively large. In addition, the average values of other phenotypic traits were the smallest in the four groups. In addition, the clustering results showed that the four groups were not clearly clustered, following the distance from provenance.

### 2.7. Excellent Clone Selection

The comprehensive multi-trait evaluation method was employed to select excellent clones. The interrelationships among phenotypic traits, determined through correlation analysis and principal component analysis, were considered during the selection process. Evaluation indices, including DBH, V, FWSC, DWSC, SWPP, TKW, CW, GR, GP, and GI, were chosen to assess the lineages. The *Q_i_* value of each lineage within a population of 40 clones was calculated. The results of the evaluation are presented in Table 5. Twelve clones with the highest *Q_i_* values were chosen, with a selection rate of 30%. Therefore, PC5, PC2, PC3, PC10, PC1, PC9, PC6, PC4, JT10, JT2, JT9, and EY6 were selected as excellent clones with outstanding comprehensive phenotypic traits, as shown in Table 6. Compared to the respective mean values of all 40 clones, the 12 selected clones exhibited higher mean values for DBH, V, FWSC, DWSC, SWPP, TKW, CW, GR, GP, and GI traits by 13.65%, 33.33%, 48.89%, 57.76%, 72.22%, 20.59%, 8.31%, 9.50%, 8.83%, and 11.06%, respectively. Moreover, the analysis of genetic gain in DBH, V, FWSC, DWSC, SWPP, and CW traits among the 12 selected excellent clones revealed an average genetic gain ranging from 4.75% to 30.71%. Among the traits, DWSC demonstrated the highest average genetic gain at 32.05%, whereas CW exhibited the smallest gain at 4.75%.

## 3. Discussion

Genetics and variation are the main components of forest tree breeding research, as well as a prerequisite and guarantee in selecting forest trees [19]. The analysis of variance is a commonly used method to evaluate the extent of trait variation [38,39,40]. The 16 phenotypic traits investigated in this study were associated with growth, cone, seed, and germination in 40 *A. cremastogyne* clones sourced from four provenances. The results of variance analysis showed that most of the phenotypic traits had highly significant differences (*p* < 0.01) or significant differences (*p* < 0.05) among and within provenances, and there were abundant variations in phenotypic traits of *A. cremastogyne*. This finding is consistent with studies conducted on other tree species, such as *Pinus sylvestris* [41], *Phoebe bournei* [42], and *Pinus wallichiana* [43]. The considerable variation observed in the phenotypic traits of *A. cremastogyne* indicates a high potential for genetic improvement. Therefore, selecting excellent trees based on phenotypic traits provides a solid foundation.

**Figure 9:**
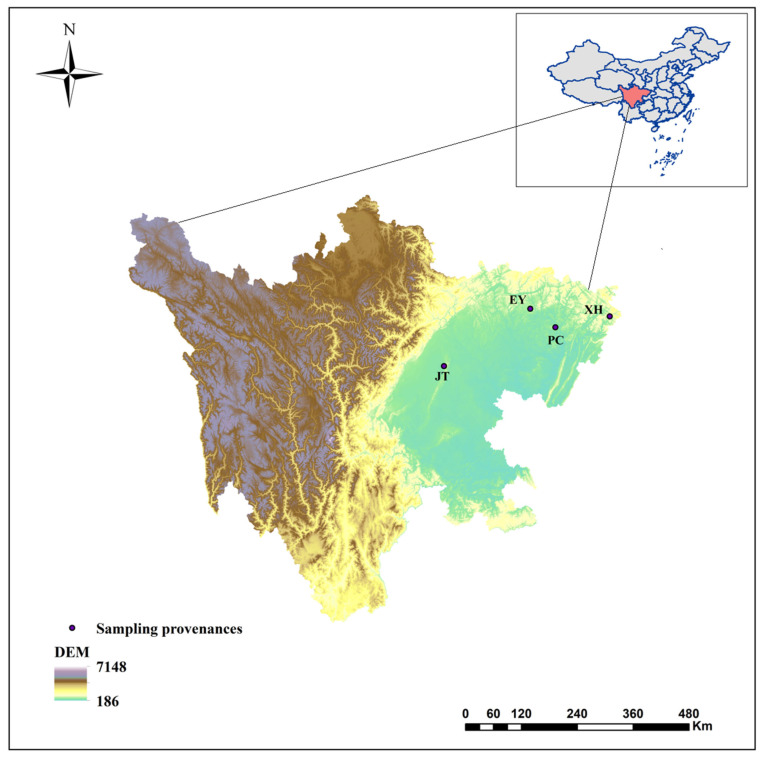
Geographical location map of 4 provenances of *A. cremastogyne*.

The phenotypic differentiation coefficient reflects the degree of phenotypic differentiation between provenances or production areas. It shows the extensive degree of plant adaptation to different environments. This study found that the average differentiation coefficient of 16 phenotypic traits of *A. cremastogyne* was 52.61%, indicating that the phenotypic variation of *A. cremastogyne* was mainly derived from among provenances. According to the phenotypic variation analysis in *A. cremastogyne*, we can clearly observe substantial differences in the phenotypic traits among different provenances. This is similar to the phenotypic diversity observed in populations of *Idesia polycarpa* [44]. Overall, the Pingchang, Jintang, and Enyang provenances of *A. cremastogyne* demonstrate superior comprehensive phenotypic performance compared to the Xuanhan provenance. This difference may be attributed to the variations in temperature across different provenance locations. Previous studies have indicated that temperature can significantly impact plant growth, with lower temperatures to some extent limiting plant development [45]. In the case of *Pinus yunnanensis*, a positive correlation between temperature and growth was observed, highlighting the growth promotion effects in higher temperature environments [46]. *A. cremastogyne* is a thermophilic and heliophilic tree species that thrives in regions with higher temperatures, which also promote its growth [4]. According to the geographic information of the different provenances of *A. cremastogyne* presented in Figure 9 and Table 7, it is evident that the Jintang, Pingchang, and Enyang provenances are located at lower altitudes, while the Xuanhan provenance is situated in the higher-altitude mountainous areas outside the Sichuan Basin. *A. cremastogyne* from the Xuanhan provenance, growing at higher altitudes in mountainous regions, receives less sunlight and experiences lower temperatures compared to the other three provenances. These factors to some extent restrict its growth. Under prolonged temperature influences, phenotypic traits of *A. cremastogyne* among different provenances have undergone varying degrees of differentiation.

In contrary to the results of this study, Guo et al. [47] conducted a study using SSR molecular markers to investigate the diversity of 175 populations of *A. cremastogyne* in Sichuan Province, and they observed higher levels of genetic variation within populations than among populations. The possible reasons for the differences observed at the molecular and macroscopic levels are that SSR markers are not influenced by the environment, but are affected by population migration or gene flow within the species, which can lead to genetic variation within the population [12]. However, phenotypic traits observed at the macroscopic level are easily influenced by environmental factors. These environmental disparities can significantly impact the expression of phenotypic traits, resulting in noteworthy variations among populations distributed in different geographical locations. However, it is important to note that SSR markers only reflect the internal DNA variations within individuals and may not necessarily be expressed in the phenotype. Therefore, relying solely on genotyping or SSR markers for genetic diversity analysis and core germplasm extraction may not effectively capture the complete genetic diversity of the species [48]. Consequently, combining phenotypic and SSR marker analysis to a certain extent can effectively reveal the genetic variation of *A. cremastogyne*. In comparison to other tree species, *A. cremastogyne* has a lower phenotypic differentiation coefficient than *P. bournei* (70.83%) [49], while this is higher than *Orchis mascula* (20.00%) [50]. It is also similar to *Juglans mandshurica* (50.31%) [51]. Provenance differences are primarily caused by gene–environment interactions under different environmental conditions [52]. This discrepancy in phenotypic coefficients between different tree species reflects the universal influence of gene-environment interactions on phenotypic variation, which is the fundamental cause of population differentiation [53]. Research has revealed that the interaction between genes and the environment (G × E) elucidates the intricate correlation between genetic traits and the growth conditions of plants. Among the observed variations in phenotypic traits, the environment has been found to play a paramount role, accounting for 80% of the observed phenotypic variation, while the genotype accounts for 10% to 15% of the phenotypic variation. Thus, the environment emerges as the primary factor impacting the observed phenotypic variations [54]. The phenotypic differentiation coefficient of *A. cremastogyne* is above the middle level, and the phenotypic variation among provenances is higher than that within provenances. This may be due to the geographical isolation among the various source areas, leading to phenotypic differentiation among the provenances of *A. cremastogyne*. It has been shown that species of the Betulaceae family are pollinated by wind [55] since, due to the limited distance of pollen transmission, different populations of *A. cremastogyne* have been geographically isolated for a long time, thereby each population gradually formed its own relatively stable population phenotypic characteristics. These factors contribute to the independent differentiation of populations and reflect the adaptation of different provenances of *A. cremastogyne* to diverse environments [56]. Variations among populations indicates the differences in geographical and reproductive isolation, highlighting the importance of intraspecific diversity [57]. Therefore, when selecting excellent clones for breeding, it is essential to consider both among and within population variations with the concern of primary sources of variation. On the other hand, the growth and distribution area of *A. cremastogyne* in Sichuan has changed significantly from the mountains around the Sichuan Basin to the hills and plains of the Yangtze River Basin, which has affected the spread of pollen and seeds in space. The gene exchange among populations is blocked, which increases the possibility of differentiation between populations. The complex geographical and climatic conditions have a great influence on the phenotypic variation of *A. cremastogyne*. It can be seen that the isolation of *A. cremastogyne* in time and space has caused abundant phenotypic trait variations to adapt to a new environment.

CV and repeatability are essential parameters used to assess genetic variation. This is beneficial to selecting excellent clones within a breeding population. These parameters provide valuable insights into the extent of variation present. The CV measures the population’s phenotypic diversity dispersion and reflects the genetic variation potential of phenotypic traits. A higher CV indicates a more extensive distribution among populations and a greater genetic variation [58]. Repeatability is an index that assesses trait stability and indicates the reliability of genotype recognition through phenotypic expression [59]. By calculating repeatability, we can determine the extent of stability in phenotypic traits and the proportion of observed phenotypic variation attributable to genetic factors. Higher repeatability values indicate a greater contribution of genetic factors to the observed variation, indicating that the phenotypic trait is more stable and reliable in repeated measurements or observations. In previous field experiments on phenotypic traits conducted by Wang et al. [60] on *Xanthoceras sorbifolium* and Liang et al. [61] on *Pinus koraiensis*, they calculated repeatability at medium to high levels. This suggests that the measured phenotypic traits are more strongly controlled by the genotype, and the phenotypic variation is relatively stable. Therefore, utilizing these phenotypic traits with higher repeatability allows for more accurate assessment of trait differences among individual trees and selection of superior individuals with desirable traits, which is of great significance for tree breeding and genetic improvement. We observed a wide range of variation in phenotypic traits, with the CV ranging from 9.41% (FSI) to 97.19% (SWPP). It was noteworthy that the coefficients of variation for CL (13.62%) and CW (14.56%) were relatively small, whereas FWSC (89.21%), DWSC (91.32%), and SWPP (97.19%) exhibited larger coefficients of variation. This indicates that CL and CW traits are more stable, while FWSC, DWSC, and SWPP showed greater variability as compared to other traits. Previous studies had shown that there may be a functional correlation between cone and seed traits in some tree species, so it is estimated that they are genetically related [62]. The overall high phenotypic variability of *A. cremastogyne* indicate a significant variation in the phenotypic traits, and the high coefficient of variation is crucial for the selection of excellent clones. The abundant phenotypic variation among clones can lead to greater genetic gain. Regarding repeatability, our findings showed repeatability values ranging from 0.36 (FSI) to 0.77 (SWPP) for each phenotypic trait, belonging to the moderate or high level (R > 0.30). The repeatability of growth traits (H, DBH, V) is similar to that of *Betula platyphylla* [63], while it is lower than that of *L. kaempferi* [64]. As for cone and germination traits, except for the repeatability value of 0.41 for SR, the repeatability of other traits exceeded 0.5. These results aligned with the findings from studies on *Pinus sylvestis* [65], although the repeatability of cone traits in our study was smaller than that of *P. koraiensis* [66]. Overall, our study demonstrated that the phenotypic traits of *A. cremastogyne* were predominantly influenced by moderate to high genetic factors. This ensured stable inheritance of these traits. Such moderate to high genetic control facilitates the selection process in breeding programs, enabling the use of fewer families for achieving a significant genetic gain [67]. The high repeatability values observed for these phenotypic traits indicate their reduced susceptibility to environmental influences, allowing for more stable inheritance and significant genetic gain. Additionally, the high repeatability of these traits is advantageous in selecting excellent clones during subsequent screening processes.

Identifying the relationship between phenotypic traits is important in forest tree breeding. Correlation analysis provides valuable insights into the relationship between different traits, which could contribute to the selection of excellent clones and the comprehensive evaluation of parents during forest improvement. In this study, the traits H, DBH, and V exhibited a strong positive correlation. There is a significant positive correlation between traits H, DBH, and V, indicating that these traits can be used both to assess the genetic variation in *A. cremastogyne* and to evaluate and select high-yielding excellent clones for genetic improvement purposes. The correlation between V and DBH was exceptionally high. This finding is similar to research on *Jatropha curcas* [68]. It further supports the notion that there is a robust correlation between volume and DBH, suggesting that the larger DBH of *A. cremastogyne* trees can increase wood production. The HUB showed a significant negative correlation with most of the phenotypic traits. This implied that, when the HUB surpassed a specific height, the longitudinal canopy size decreased, leading to reduced nutrient acquisition through photosynthesis and negative effects on other traits.

There was a highly significant positive correlation among growth, cone, and seed traits, likely attributable to growth and reproductive competition during nutrient redistribution processes. Furthermore, most cone and seed traits displayed significant positive correlations, which was consistent with previous research findings [69]. The significance of seed traits indicated that FWSC, DWSC, SWPP, TKW, CL, and CW were the primary factors influencing the efficiency of seed trait measurement. Both cone and seed traits could be utilized for subsequent evaluation and selection. Seed weight is an essential indicator of seed quality and positively correlates with seed germination rate [70,71]. Seed germination and emergence depend on embryo size and seed nutrients. Seeds with higher thousand-grain weight contain more stored nutrients, facilitating embryo growth and seed germination. Mughal and Thapliyal. [72] found that seed germination in *Cedrus deodara* was related to seed/cone size, and higher germination rates. This study showed a significant positive correlation between TKW GR, GP, and GI. However, these findings were differed from a previous work on *A. cremastogyne* by Li et al. [73]. It was probably attributed to the different genetic characteristics among clones in different provenances or variations in seed maturity, collection time, and storage methods.

The PCA is a classical multivariate value technology [74]. It utilizes the relationships between variables to eliminate redundant information through projection and dimensionality reduction, reducing the complexity of variables to one or a few principal components. In this study, the cumulative contribution rate of the three principal components for *A. cremastogyne* was 79.18%, which is comparable to oak [75], while it is higher than willow [76] and *Carthamus tinctorius* [77]. The high cumulative variation in PCA can be attributed to the strong multicollinearity between traits and their significant correlations [78]. Specifically, PCA1 was primarily associated with growth, cone, and seed traits, PCA2 was associated with seed germination traits, and PCA3 was associated with cone seed traits. The findings of this study demonstrated that the phenotypic diversity of *A. cremastogyne* was influenced by a combination of growth traits, cone traits, seed traits, and germination traits. This observation is consistent with previous research on *Arenga pinnata* [79], indicating a stable correlation among the analyzed phenotypic traits and providing a basis for identifying excellent clones.

A clustering dendrogram of the 40 clones was constructed based on PCA, and the results found that the first and second groups exhibited better comprehensive phenotypic traits than the third and fourth groups, indicating that excellent clones were likely to be selected from the first and second groups after the evaluation and screening of the parents. The performance of phenotypic traits within each classification group further supported the selection of excellent clones. It is essential to highlight that the clustering results do not align perfectly with the geographical distance between provenances, which is similar to the findings of *P. yunnanensis* [46]. This observation may be attributed to factors such as topography, soil conditions, altitude, and vegetation type [80]. Moreover, it further supports the idea that geographical isolation contributes to the independent differentiation of phenotypic traits among *A. cremastogyne* provenances.

Phenotypic variation is the fundamental guarantee for excellent clones. Trees have abundant genetic variation, allowing selective breeding to promote the application of superior germplasm. Genetic improvement of trees plays a crucial role in ensuring stable inheritance for the offspring. There are multiple methods for selecting excellent clone lines, such as indicator selection [81] and PCA evaluation [82]. However, the method of comprehensive evaluation based on calculating *Q_i_* from multiple traits is considered more accurate. To avoid reducing the genetic gain of individual traits, selecting appropriate traits as evaluation indicators is necessary [61]. This study employed the combination of correlation analysis and PCA to choose ten closely related traits (DBH, V, FWSC, DWSC, SWPP, TKW, CW, GR, GP, GI) as the evaluation index for the *Q_i_* value. These traits represented the growth, cone, seed, and germination characteristics of *A. cremastogyne*. Twelve excellent clones were selected based on their *Q_i_* values. The average genetic gains for growth traits of DBH and V were 4.78% and 9.10%, respectively. They were slightly lower than that of *P. koraiensis* [83] and *L. kaempferi* [84]. The average genetic gains for cone and seed traits (FWSC, DWSC, SWPP, TKW, CW) were 22.38%, 32.05%, 25.52%, 8.31%, and 6.72%, respectively, which were greater than those of *P. koraiensis* [85]. Similarly, the average genetic gains for germination traits (GR, GP, GI) were 8.94%, 12.57%, and 11.55%, respectively, which were larger than those of *Cunninghamia lanceolata* [86]. These variations in genetic gain can be attributed to different tree species, selection intensity, and growth environment factors. Significantly, the average genetic gain of FWSC, DWSC, and SWPP traits was higher than that of other traits, indicating the superior performance of seed traits in *A. cremastogyne*. In general, the selected excellent clones with high genetic gain will meet the current market demand for high-quality *A. cremastogyne* seeds. Moreover, it will contribute to the improvement of the structure of *A. cremastogyne* seed orchards and the optimization of breeding material selection.

## 4. Materials and Methods

### 4.1. Study Sites and Materials

The study was conducted at the National Primary Clone Seed Orchard of *A. cremastogyne*, located in Pingchang County (PC), Sichuan Province, Southwest China (107°25′ E, 31°34′ N). The seed orchard is located in the subtropical monsoon climate, with an average altitude of 540 m. The average annual temperature is 16.8 °C, the annual average rainfall 1213 mm, and the soil in the experimental area is slightly acidic and moderately fertile. In the spring of 2017, the cuttings of *A. cremastogyne* were collected from four provenances of Pingchang County (PC), Enyang District (EY), Jintang County (JT), and Xuanhan County (XH) in Sichuan Province (Figure 9). To minimize the influence of microhabitat, the harvested branches were randomly grafted in the seed orchard as garden materials. The seed orchard was divided into three large areas, with 2 m × 3 m spacing among individual plants and cuttings. This experiment employed a completely randomized experimental design, in which 40 *A. cremastogyne* clones from four provenances within the seed orchard were selected as experimental materials, with three individual plants randomly chosen from each clone for subsequent phenotypic measurements. For this study, 40 *A. cremastogyne* clones were selected from the seed orchard, and three individual plants were chosen within each clone for phenotypic traits analysis. The phenotypic traits of all the selected clones were measured between November 2022 and December 2022. The basic information on the experimental materials is provided in Table 7.

### 4.2. Index Measurement Calculation

The height of trees (*H*) was measured using a height measuring rod with an accuracy of 0.1 m. The diameter at breast height (DBH) was measured 1.3 m above the ground level using a DBH tape measure with an accuracy of 0.1 cm. The height under the branches (HUB) was determined by measuring the height of the first living branch using a tape measure with an accuracy of 0.1 m. The volume (*V*) was calculated based on the following formula [87]:

(1) $$V=5.275071\times 10^{-5}\times D^{1.9450324}\times H^{0.9388533}$$

where *V* is volume, *D* is the diameter at breast height, and *H* is the height of trees.

The fresh weight of cones (FWSC), dry weight of cones (*DWSC*), and seed weight per plant (*SWPP*) were measured using an electronic balance with a precision of 0.01 kg. To determine the cone length (*CL*) and cone width (*CW*), a vernier caliper with an accuracy of 0.01 mm was employed. For each plant, a random selection of 20 cones was made, resulting in a total of 60 cones measured per clone. The thousand kernel weight (TKW) was determined using the quartering method, with a precision of 0.01 g. The fruit shape index (*FSI*) and seed rate (*SR*) were calculated according to the following formulas:

(2) $$FSI=\frac{CL}{CW}$$

where *FSI* is the fruit shape index, *CL* is the cone length, and *CW* is the cone width.

(3) $$SR=\frac{SWPP}{DWSC}\times 100\%$$

where *SR* is the seed rate, *SWPP* is the seed weight per plant, and *DWSC* is the dry weight of single cone.

The seed germination test was conducted in an incubator, and the germination rate (*GR*), germination potential (*GP*), and germination index (*GI*) were calculated the following formula [88]:

(4) $$GR=\frac{N}{M}\times 100\%$$

where *GR* is the germination rate, *N* is the number of normal germinating seeds, and *M* is the total number of seeds.

(5) $$GP=\frac{K}{M}\times 100\%$$

where GP is the germination potential, *K* is the number of normal germinating seeds at peak times, and *M* is the total number of seeds.

(6) $$GI=\frac{\sum Gt}{Dt}\times 100\%$$

where *GI* is the germination index, *Gt* is the germination rate at time (*t*), and *Dt* is the duration of the germination test.

### 4.3. Statistical Analyses

According to the results of nested analysis of variance, which revealed differences in the phenotypic traits among different provenances of *A. cremastogyne*, the Duncan multiple comparison method was employed to compare the means and standard deviations of the phenotypic traits, and relevant genetic erosion parameters were calculated. Pearson correlation coefficient was used to analyze the correlation among the various phenotypic traits. Principal component analysis was conducted to reduce the dimensionality of the phenotypic trait variables, and based on the results of principal component analysis the Euclidean distance method was used to cluster the clone. The experimental data were summarized using Microsoft Office Excel 2019, and the nested variance analysis, multiple comparisons, correlation analysis, principal component analysis, and cluster analysis were conducted using IBM SPSS Statistics 27, and the graphs were generated using OriginPro2022b. For the data analysis, nested variance analysis was employed, and the linear model of variance analysis according to the following statistical model [89]:

(7) $$Y_{ijk}=\mu +P_{i}+C{\left(P\right)}_{i(j)}+\varepsilon_{ijk}$$

where $Y_{ijk}$ is an individual plant observation; μ is the overall mean; $P_{i}$ is the effect of among provenances; $C{\left(P\right)}_{i(j)}$ is the clone within provenances effect; and $\varepsilon_{ijk}$ is the random error.

The phenotypic coefficient of variation was calculated as follows:

(8) $$CV=\frac{SD}{\bar{X}}\times 100\%$$

where CV is the coefficient of variation, SD is the standard deviation of the mean value of a trait, and $\bar{X}$ is the mean value of trait.

The phenotypic differentiation coefficient was calculated according to the following formula [90]:

(9) $$V_{st}=\frac{\sigma_{t/s}^{2}}{\left(\sigma_{t/s}^{2}+\sigma_{s}^{2}\right)}$$

where is $V_{st}$ the phenotypic differentiation coefficient; $\sigma_{t/s}^{2}$ is the variance component among provenances, and $\sigma_{s}^{2}$ is the variance component within provenances.

The repeatability (*R*) for phenotypic trait was calculated according to the following formula [91]:

(10) $$R=1-\frac{1}{F}$$

where *R* is the repeatability, and *F* is the value in nested analysis of variance.

The correlation coefficients $r_{p(xy)}$ was calculated according to the following formulas [40]:

(11) $$r_{p(x,y)}=\frac{{Cο\nu }_{p(x,y)}}{\sqrt{\sigma_{px}^{2}\cdot \sigma_{py}^{2}}}$$

where $r_{p(xy)}$ represents the correlation coefficients, and ${Cο\nu }_{p(x,y)}$ is the phenotypic covariance between the traits *x* and *y*; $\sigma_{px}^{2}$ and $\sigma_{py}^{2}$ denote the phenotypic variance of traits *x* and *y*, respectively.

The multi-trait comprehensive evaluation was calculated using the following formula [92]:

(12) $$Q_{i}=\sqrt{\sum_{j=1}^{n}a_{i}}$$

where $Q_{i}$ is the comprehensive evaluation value of each clone; $a_{i}=X_{ij}/X_{jmax}$; $X_{ij}$ is the mean value of a given trait; $X_{jmax}$ is the maximum mean value of the trait; and *n* is the number of the number of the traits.

The genetic gain was calculated according to the following formula [93]:

(13) $$∆G=\frac{R\cdot S}{\bar{X}}$$

where ∆G is the genetic gain; *R* is the repeatability of the trait; *S* is the selection bias; and $\bar{X}$ is the overall mean value of the trait.

## 5. Conclusions

*A. cremastogyne*, as one of the main fast-growing timber species in Southwest China, has significant economic and ecological value. Therefore, it is crucial to study and evaluate the genetic variation of its phenotypic traits the growth, cone, seed, and germination traits of 40 *A. cremastogyne* clones were measured and analyzed in this work. The results demonstrated that the phenotypic traits of *A. cremastogyne* were predominantly governed by genetic control, exhibiting substantial genetic variation. Moreover, the observed phenotypic traits variation primarily came from among provenances. Using correlation analysis and principal component analysis, and ten phenotypic traits serving as comprehensive evaluation indicators, PC5, PC2, PC3, PC10, PC1, PC9, PC6, PC4, JT10, JT2, JT9, and EY6 were selected as excellent clones. These excellent clones can be used as breeding materials for the upgrading of the *A. cremastogyne* seed orchard. This selection process aimed to enhance the reliability and genetic gain of improved varieties, and provide a theoretical foundation for the genetic improvement and the breeding of excellent resources of *A. cremastogyne*.

## Figures and Tables

**Table 1:** Variance analysis of phenotypic traits of *A. cremastogyne*.

Traits	MS (df)			F Value	
	Among Provenances	Within Provenances	Error	Among Provenances	Within Provenances
DBH	52.289 (3)	1.722 (36)	0.98 (80)	53.48 **	1.76 *
H	20.259 (3)	1.462 (36)	0.58 (80)	34.62 **	2.50 **
V	0.002 (3)	0.000 (36)	0.000 (80)	36.14 **	1.55
HUB	0.696 (3)	0.101 (36)	0.042 (80)	16.54 **	2.40 **
FWSC	6.706 (3)	1.219 (36)	0.507 (80)	13.24 **	2.41 **
DWSC	2.676 (3)	0.405 (36)	0.132 (80)	20.24 **	3.07 **
SWPP	0.736 (3)	0.058 (36)	0.028 (80)	26.24 **	2.07 **
TKW	0.173 (3)	0.020 (36)	0.009 (80)	18.33 **	2.09 **
CL	42.483 (3)	9.651 (36)	4.285 (80)	9.91 **	2.25 **
CW	25.302 (3)	3.420 (36)	0.775 (80)	32.66 **	4.42 **
CL × CW	26,131.131 (3)	4518.274 (36)	1335.769 (80)	19.56 **	3.38 **
FSI	0.169 (3)	0.047 (36)	0.020 (80)	8.30 **	2.30 **
SR	1608.128 (3)	175.433 (36)	104.043 (80)	15.46 **	1.69 *
GR	849.025 (3)	308.387 (36)	95.200 (80)	8.92 **	3.24 **
GP	696.195 (3)	268.846 (36)	96.262 (80)	7.23 **	2.79 **
GI	5.382 (3)	1.752 (36)	0.567 (80)	9.54 **	3.10 **

MS: mean squares; df: degrees of freedom; F: F value; *: *p* < 0.05, **: *p* < 0.01. DBH: diameter at breast height; H: height of trees; V: volume; HUB: height under the branches; FWSC: fresh weight of single cone; DWSC: dry weight of single cone; SWPP: seed weight per plant; TKW: thousand kernel weight; CL: cone length; CW: cone width; CL × CW: cone length × cone width; FSI: fruit shape index; SR: seed rate; GR: germination rate; GP: germination potential; GI: germination index.

**Table 2:** The Mean, Min, Max, Scope and Standard Deviation (SD) of phenotypic traits.

Traits	Unit	Mean	Min	Max	Range	SD
DBH	cm	8.50	5.10	12.80	7.70	1.56
H	m	7.57	5.14	10.24	5.10	1.12
V	m^3^	0.02	0.01	0.06	0.05	0.01
HUB	m	1.23	0.53	1.95	1.42	0.28
FWSC	kg	1.05	0.19	3.86	3.67	0.94
DWSC	kg	0.58	0.11	2.84	2.74	0.53
SWPP	kg	0.24	0.06	0.96	0.91	0.23
TKW	g	0.51	0.11	0.85	0.74	0.13
CL	cm	19.25	14.56	27.84	13.28	2.62
CW	cm	10.17	6.89	15.23	8.34	1.48
CL×CW	cm	198.74	104.18	392.63	288.45	54.07
FSI	—	1.90	1.51	2.63	1.12	0.18
SR	%	40.59	14.08	72.65	58.57	12.72
GR	%	53.87	17.50	90.50	73.00	13.73
GP	%	46.64	12.60	85.50	72.90	13.38
GI	—	3.50	1.16	6.28	5.12	1.04

The abbreviation of phenotypic traits is shown in Table 2.

**Table 3:** PCA of phenotypic traits of *A. cremastogyne*.

Traits	Component		
	PCA1 (Y_1_)	PCA2 (Y_2_)	PCA3 (Y_3_)
DBH	0.86	−0.080	−0.42
H	0.68	−0.28	−0.52
V	0.86	−0.15	−0.42
HUB	−0.67	−0.09	−0.04
FWSC	0.85	−0.12	0.29
DWSC	0.85	−0.11	0.37
SWPP	0.89	−0.03	0.25
TSW	0.77	0.36	0.01
CL	0.75	−0.27	0.40
CW	0.87	−0.22	0.07
CL × CW	0.86	−0.26	0.27
FSI	−0.44	0.01	0.50
SR	0.53	0.36	−0.27
GR	0.26	0.95	0.02
GP	0.24	0.94	0.08
GI	0.24	0.94	0.03
Eigenvalues	7.95	3.26	1.46
Contribution%	49.69	20.36	9.13
Cumulative%	49.69	70.05	79.18

The abbreviation of phenotypic traits is shown in Table 1.

**Table 4:** Mean value of phenotypic traits in each group.

Traits	Groups			
	Ⅰ	II	III	IV
DBH (cm)	10.48	8.94	7.89	5.75
H (m)	8.17	7.85	7.14	6.21
V (m³)	0.04	0.03	0.02	0.01
HUB (m)	0.95	1.16	1.33	1.58
FWSC (kg)	3.31	1.12	0.68	0.38
DWSC (kg)	2.09	0.59	0.37	0.27
SWPP (kg)	0.84	0.26	0.13	0.08
TSW (g)	0.69	0.54	0.46	0.44
CL (cm)	24.03	20.00	17.76	15.60
CW (cm)	13.91	10.51	9.40	7.45
CL × CW (cm)	335.28	211.64	167.59	116.37
FSI	1.73	1.91	2.10	1.89
SR (%)	43.36	44.48	34.20	31.37
GR (%)	49.43	57.93	46.60	58.85
GP (%)	42.88	50.63	40.13	50.17
GI	3.17	3.82	3.03	3.62

The abbreviation of phenotypic traits is shown in Table 1.

**Table 5:** *Q_i_* value of 40 clones.

Clone	*Q_i_*	Clone	*Q_i_*	Clone	*Q_i_*	Clone	*Q_i_*
PC5	3.289	JT9	2.958	EY9	2.818	JT1	2.596
PC2	3.153	EY6	2.955	EY7	2.810	XH1	2.567
PC3	3.111	JT3	2.894	EY4	2.799	XH9	2.515
PC10	3.094	EY8	2.856	JT6	2.766	XH3	2.489
PC1	3.094	PC7	2.853	JT4	2.752	XH5	2.426
PC9	3.085	JT8	2.846	JT7	2.715	XH4	2.426
PC6	3.054	EY10	2.840	JT5	2.702	XH6	2.421
PC4	3.004	PC8	2.824	EY3	2.676	XH8	2.123
JT10	2.979	EY2	2.820	EY5	2.625	XH10	1.965
JT2	2.971	EY1	2.818	XH2	2.601	XH7	1.790

The abbreviation of phenotypic traits is shown in Table 1.

**Table 6:** Genetic gain of excellent asexuality at 30% selection rate.

Clone	Genetic Gain (%)									
	DBH	V	FWSC	DWSC	SWPP	TKW	CW	GR	GP	GI
PC5	10.24	16.27	25.52	51.19	17.38	5.44	3.35	27.41	26.96	23.16
PC2	13.50	24.74	28.42	63.02	36.65	1.19	6.30	8.46	11.86	8.52
PC3	6.16	10.93	20.51	30.62	19.11	4.06	8.17	5.00	6.40	7.19
PC10	2.85	8.86	22.35	10.91	17.25	17.90	4.47	6.60	9.91	14.15
PC1	4.39	7.05	16.48	5.13	6.83	7.63	7.89	3.29	23.63	10.91
PC9	2.22	3.67	12.55	1.05	8.32	9.66	5.99	8.00	3.92	5.20
PC6	3.00	5.63	8.45	20.80	23.44	5.68	0.77	5.93	20.02	3.83
PC4	1.00	3.22	33.77	44.78	42.00	4.46	2.25	13.28	16.29	23.41
JT10	0.51	2.69	8.77	31.73	40.19	16.36	3.28	10.26	13.90	16.43
JT2	8.27	15.69	27.54	37.55	32.40	7.53	2.48	15.71	14.45	19.72
JT9	1.34	2.23	18.88	41.00	32.66	4.39	7.27	15.19	16.47	17.02
EY6	7.96	17.24	39.17	42.11	35.96	10.90	11.86	3.08	3.37	4.02
mean	4.86	9.18	20.29	30.71	25.11	7.66	4.75	10.83	14.89	13.59

The abbreviation of phenotypic traits is shown in Table 1.

**Table 7:** The situation for different tested provenances of *A. cremastogyne* clones.

Provenances	Clone Number	Longitude	Latitude	Average Altitude (m)	Average Annual Temperature (°C)	Average Annual Rainfall (mm)
PC	PC1–PC17	107°03′ E	31°56′ N	488	16.8	1213
EY	EY1–EY6	106°49′ E	31°94′ N	580	17.8	1050
JT	JT1–JT7	104°48′ E	30°95′ N	456	16.9	920
XH	XH1–XH10	108°27′ E	31°68′ N	625	16.8	1230

The abbreviation of provenances is shown in Figure 1.

## Data Availability

Not applicable.
